# Percutaneous transhepatic cholangial drainage or antibiotic therapy worsens response to immunotherapy in advanced cholangiocarcinoma

**DOI:** 10.1186/s12885-023-11128-2

**Published:** 2023-07-13

**Authors:** Qingyu Huang, Fuhao Wang, Xiang Zhang, Jing Liu, Xue Dou, Rui Feng, Kunli Zhu, Shumei Jiang, Yun Zhang, Jinbo Yue

**Affiliations:** 1 Department of Radiation Oncology, Shandong Cancer Hospital and Institute, Shandong First Medical University, Shandong Academy of Medical Sciences, Jinan, 250117 China; 2 School of Clinical Medicine, Weifang Medical University, Weifang, 261053 China

**Keywords:** Percutaneous transhepatic cholangial drainage, Antibiotic, Immunotherapy, Cholangiocarcinoma, Microbiota

## Abstract

**Background:**

Bile duct obstruction is a common issue for patients with advanced cholangiocarcinoma (CCA). Percutaneous transhepatic cholangial drainage (PTCD) is often required to relieve the obstruction. However, PTCD may alter the intestinal microbiota, which can affect the efficacy of immunotherapy. Antibiotics (ATB) can also have significant immunomodulatory effects by perturbing the gut microbiota. Therefore, this study aimed to investigate whether PTCD or ATB therapy is associated with overall survival (OS) or progression-free survival (PFS) in patients with advanced CCA receiving first-line chemotherapy plus immune checkpoint blockade (ICB) in clinical practice. We also explored whether the gut microbiota changes after receiving PTCD.

**Methods:**

We conducted a single-center retrospective analysis of PTCD and ATB therapy in patients with advanced CCA. PTCD was performed before ICB initiation, and ATB was administered within 1 month before and 6 weeks after ICB initiation. Our primary outcomes were PFS and OS. Moreover, we used 16s rRNA sequencing to analyze fecal and bile samples obtained from patients who underwent PTCD.

**Results:**

In total, 107 patients with CCA were included. Among patients who did not undergo PTCD, ICB plus chemotherapy significantly improved OS vs. chemotherapy alone (hazard ratio [HR] 0.21, 95% confidence interval [CI] 0.09–0.45, *p* < 0.0001). PFS was also significantly improved in patients who received ICB plus chemotherapy compared with chemotherapy alone (HR 0.36, 95% CI 0.16–0.80, *p* = 0.0024). However, ICB plus chemotherapy did not improve survival compared with chemotherapy alone among patients who received PTCD. Overall changes in the fecal microbiota of patients after PTCD involved significant reductions in which *Escherichia − Shigella*.

**Conclusions:**

The use of ATB or PTCD in patients with CCA receiving ICB was associated with worse OS compared with chemotherapy alone, and PTCD affects the gut microbiota. *Escherichia − Shigella* was significantly reduced in feces of patients after PTCD.

**Supplementary Information:**

The online version contains supplementary material available at 10.1186/s12885-023-11128-2.

## Background

Cholangiocarcinoma (CCA) is the second most common malignant tumor of the liver after hepatocellular carcinoma [1, 2]. So far, the only potential curative treatment for CCA is surgical resection [3]. However, in most cases the disease is diagnosed at an advanced stage and patients are given only palliative treatment [4, 5]. The current global standard first-line chemotherapy for advanced CCA is gemcitabine and a platinum compound [6–8]. In recent years, immune checkpoint blockade (ICB) have achieved promising results in the treatment of some malignant tumors [9]. In several clinical trials, it has been confirmed that ICB plus chemotherapy can improve overall survival in patients with advanced CCA [10–13].

Patients with CCA often experience bile duct obstruction [14]. Interventional surgery such as percutaneous transhepatic cholangial drainage (PTCD) is often required to relieve the obstruction before systemic antitumor therapy can be used. The gut microbiota can regulate antitumor immunity and affect the efficacy of immunotherapy [15]. Antibiotics (ATB) are known to have profound immunomodulatory effects, primarily through perturbation of the gut microbiota [16, 17]. Decreased bile may contribute to gut microbiota dysbiosis, including lower diversity and significant structural segregation [18, 19]. Bile is drained from patients via PTCD, and gut microbiota may also be altered.

We hypothesized that bile drainage could potentially alter the intestinal microbiota of patients, thereby impacting the effectiveness of immunotherapy. Our objective was to collect evidence supporting the potential enhancement of immunotherapy efficacy by modulating the intestinal microbiota in patients with CCA. To accomplish this, we conducted preliminary studies to investigate the impact of PTCD or ATB on the efficacy of first-line ICB combined with chemotherapy in patients with advanced CCA. Additionally, we examined the changes in intestinal flora before and after PTCD.

## Methods

### Study population

After Institutional Review Board approval, we retrospectively identified patients with advanced CCA who received any ICB plus chemotherapy or chemotherapy alone for first-line treatment at Shandong Cancer Hospital from January 2019 through January 2022. Follow-up was completed by July 2022. All patients had stage IV unresectable disease and none received adjuvant or neoadjuvant therapy. Patients who received at least two cycles of ICB as a part of standard-of-care treatment were included; data on PTCD use before ICB initiation or systemic ATB use (inpatient or outpatient; oral or intravenous; any duration) within 1 month before and 6 weeks after the first dose of ICB were reviewed; and only patients surviving at least 6 weeks after the first ICB dose were included in the analysis. The exclusion criteria were as follows: ECOG score > 1 when receiving PTCD or ATB; PTCD for biliary obstruction after treatment had begun; The use of systemic antibiotics or probiotics within 3 months.

The highest value of direct bilirubin during admission was recorded to assign patients into one of two groups according to median value to understand the effect of bilirubin on survival. Progression-free survival (PFS) was defined as the time from the first dose of ICB to disease progression or death, as assessed according to the Response Evaluation Criteria in Solid Tumors (version 1.1). Overall survival (OS) was measured as time from first dose of ICB to death. Data were collected according to the Declaration of Helsinki principles.

### Stool and bile sample collection

To investigate the changes in intestinal flora of patients before and after PTCD, nine of 24 patients received PTCD who were able to provide available stool and bile samples both before and after the PTCD procedure were utilized for microbiological data analysis. Stool and bile samples were collected at baseline (before or on the day of PTCD) and after PTCD (1 week after PTCD) using collection tubes. Stool and bile samples were immediately frozen at -80 °C. Patients did not receive any systemic antibiotics or probiotics within 3 months before sample collection.

### 16 S rRNA gene sequencing

Stool and bile samples were collected at baseline (before or on the day of PTCD) and after PTCD (1 week after PTCD); DNA was extracted and isolated with lysis buffer and mechanical bead-beating and purified by using a QIAamp DNA Stool Mini Kit (Qiagen, Germany). The V4 variable region of the 16S rRNA gene was amplified by PCR, and the primer sequences were 515F (5’-GTGCCAGCMGCCGCGGTAA-3’) and 806R (5’-GGACTACHVGGGTWTCTAAT-3’) modified with specific barcodes. Sequencing libraries were generated with the Ion Plus Fragment Library Kit (48 rxns, Thermo Scientific). Library quality was assessed by using a Qubit 2.0 Fluorometer (Thermo Scientific). Purified PCR products were prepared for amplicon sequencing using the IonS5 TMXL platform (Thermo Fisher) and 400-bp single-end reads were generated.

### Raw data processing

Majorbio Bio-pharm Technology Co., Ltd. quality-filtered raw FASTQ files by using Trimmomatic software and merged files by using FLASH software following the standard criteria. Operational taxonomic units (OTUs) were clustered with a 97% similarity cutoff using UPARSE Version 7.1 (http://drive5.com/uparse/) with a novel ‘greedy’ algorithm that simultaneously performs chimera filtering and OTU clustering. The taxonomy of each 16 S rRNA gene sequence was analyzed by using the Ribosomal Database Project (RPD) Classifier algorithm (http://rdp.cme.msu.edu/) against the Silva database using a 70% confidence threshold.

### Statistical analysis

Survival curves were calculated by the Kaplan–Meier method and compared with log–rank tests. The LEfSe package was used to draw the histogram of the LDA value distribution of significantly different species to show the significantly enriched species in each group and their importance. Results were visualized using the ggplot2 R-package (http://ggplot2.org). Descriptive statistics were used to analyze baseline characteristics. A random forest classifier model was constructed to determine which features were important in distinguishing pre- and post-PTCD groups. The area under the curve of the receiver-operating characteristic (ROC) curve was used as the measure of model performance. Box plots were generated with Wilcoxon tests. A *p* value of < 0.05 was considered to be statistically significant. All statistical data were analyzed with GraphPad 9.0 software and R (version 4.1.3, R Foundation for Statistical Computing, Vienna, Austria).

## Results

### Patient characteristics

This retrospective analysis included 107 patients with CCA. The mean patient age was 60 years (range 32–77 years). ICB plus chemotherapy was administered in 65 patients and chemotherapy alone in 42 patients. Twenty-four patients (22%) received PTCD before treatment, 13 in the ICB plus chemotherapy group and 11 in the chemotherapy-only group. Twenty-two patients received ATB 1 month before and 6 weeks after ICB initiation, 15 in the ICB plus chemotherapy group and 7 in the chemotherapy-only group. Complete demographic and clinical data are shown in Table 1.

**Table 1:** Patient characteristics

Characteristic	No. (%)
	(n = 107)
Median age, y (range)	60 (32–77)
Sex	
Female	42 (39.3)
Male	65 (60.7)
Tumor location	
Intrahepatic bile ducts	50 (46.7)
Extrahepatic bile ducts	57 (53.3)
Treatment	
ICB + chemotherapy	65 (60.7)
Chemotherapy	42 (39.3)
ICB	
Durvalumab	4 (3.7)
Camrelizumab	26 (24.3)
Tislelizumab	8 (7.5)
Sintilimab	27 (25.2)
Chemotherapy*	107 (100)
Gemcitabine + oxaliplatin	49 (45.8)
Gemcitabine + cisplatin	58 (54.2)
PTCD	
No	83 (77.6)
Yes	24 (22.4)
ATB	
No	85 (79.4)
Yes	22 (20.6)
ECOG score	
0–1	103 (96.3)
2	4 (3.7)

ATB, antibiotic; ECOG, Eastern Cooperative Oncology Group; ICB, immune checkpoint blockade; PTCD, percutaneous transhepatic cholangial drainage

*Gemcitabine was given at a dose of 1000 mg/m^2^ on day 1 and day 8; cisplatin at a dose of 25 mg/m^2^ was given on day 1 and day 8; oxaliplatin given at a dose of 100 mg/m^2^ was given on day 1. Treatment was repeated every 3 weeks

### ICB plus chemotherapy vs. chemotherapy

**Fig. 1:**
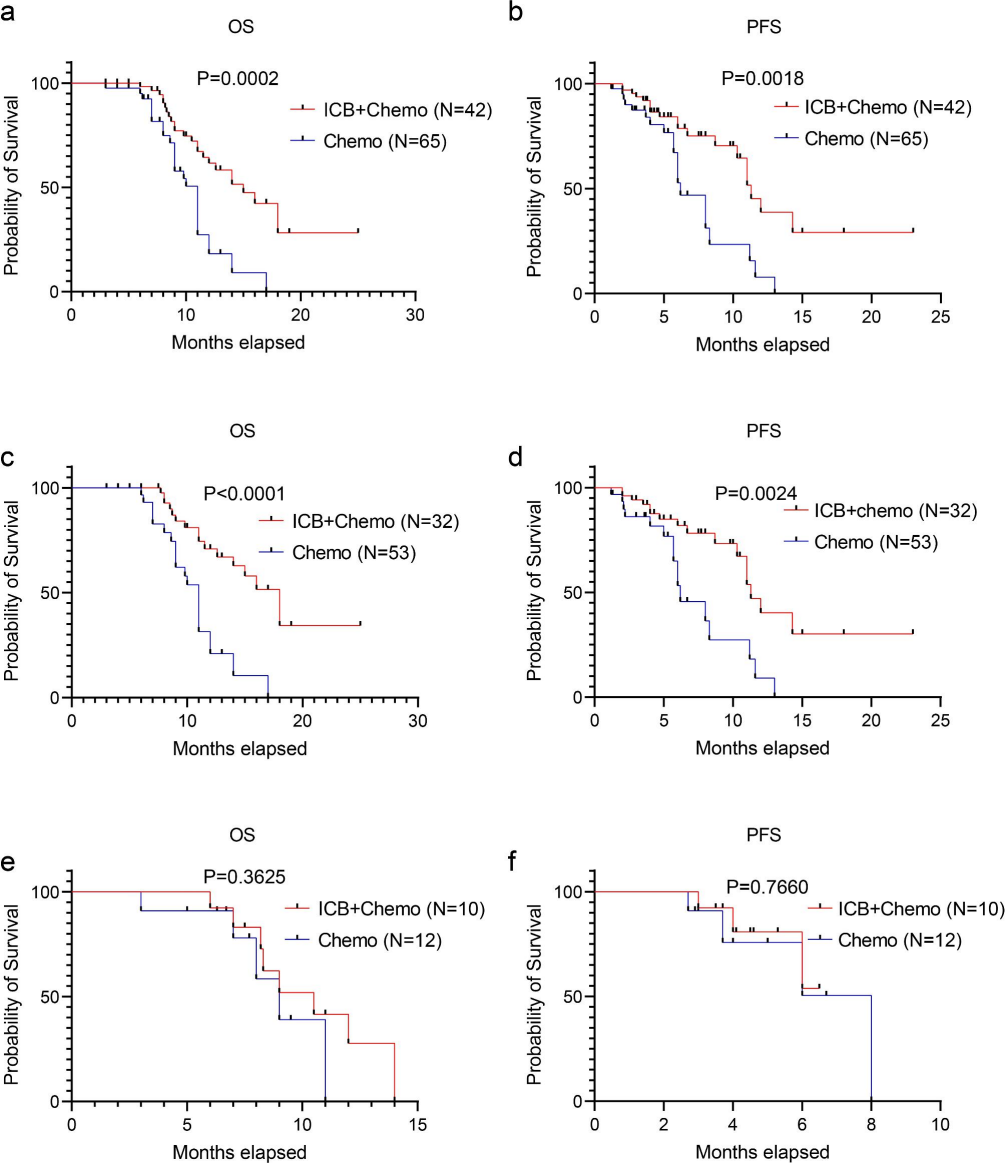
Kaplan–Meier curves of OS (**a**) and PFS (**b**) for immune checkpoint blockade (ICB) plus chemotherapy vs. chemotherapy alone in patients with advanced cholangiocarcinoma. OS (**c**), PFS (**d**) in patients who did not undergo percutaneous transhepatic cholangial drainage (PTCD). OS (**e**) and PFS (**f**) for ICB plus chemotherapy and chemotherapy alone in patients who underwent PTCD. ICB refers to immune checkpoint blockade. Chemo refers to chemotherapy

The median OS time and median PFS time for the ICB-plus-chemotherapy group were 15.0 months (95% CI 11.5–18.5) and 11.0 months (95% CI 9.8–12.8), respectively, and the median OS time and median PFS time for the chemotherapy-alone group was 12 months (95% CI 9.9–12.1) and 6.2 months (95% CI 4.9–7.5), respectively. Both OS time and PFS time for patients in the ICB-plus-chemotherapy group were significantly longer than OS time and PFS time in patients treated with chemotherapy alone (all *p* < 0.05, Fig. 1a-b). In patients who did not undergo PTCD, ICB plus chemotherapy led to significantly improved OS vs. chemotherapy alone (hazard ratio [HR] 0.21, 95% CI 0.09–0.45, *p* < 0.0001), and PFS was also significantly improved in patients who received ICB plus chemotherapy (HR 0.36, 95% CI 0.16–0.80, *p* = 0.0024) (Fig. 1c-d). However, in patients who underwent PTCD, ICB plus chemotherapy did not improve survival compared with chemotherapy alone (Fig. 1e-f). Multivariate analysis revealed that immunotherapy conferred a protective effect (HR = 0.340, P<0.001) on overall survival, while the administration of PTCD posed a risk (HR = 2.840, P = 0.003, HR, hazard ratio) to overall survival in all patients (Supplementary Table 1). Moreover, PTCD emerged as a risk factor (HR = 4.321, P = 0.003) for overall survival specifically in patients receiving ICB in combination with chemotherapy (Supplementary Table 2).

### Effect of PTCD on antitumor therapy

**Fig. 2:**
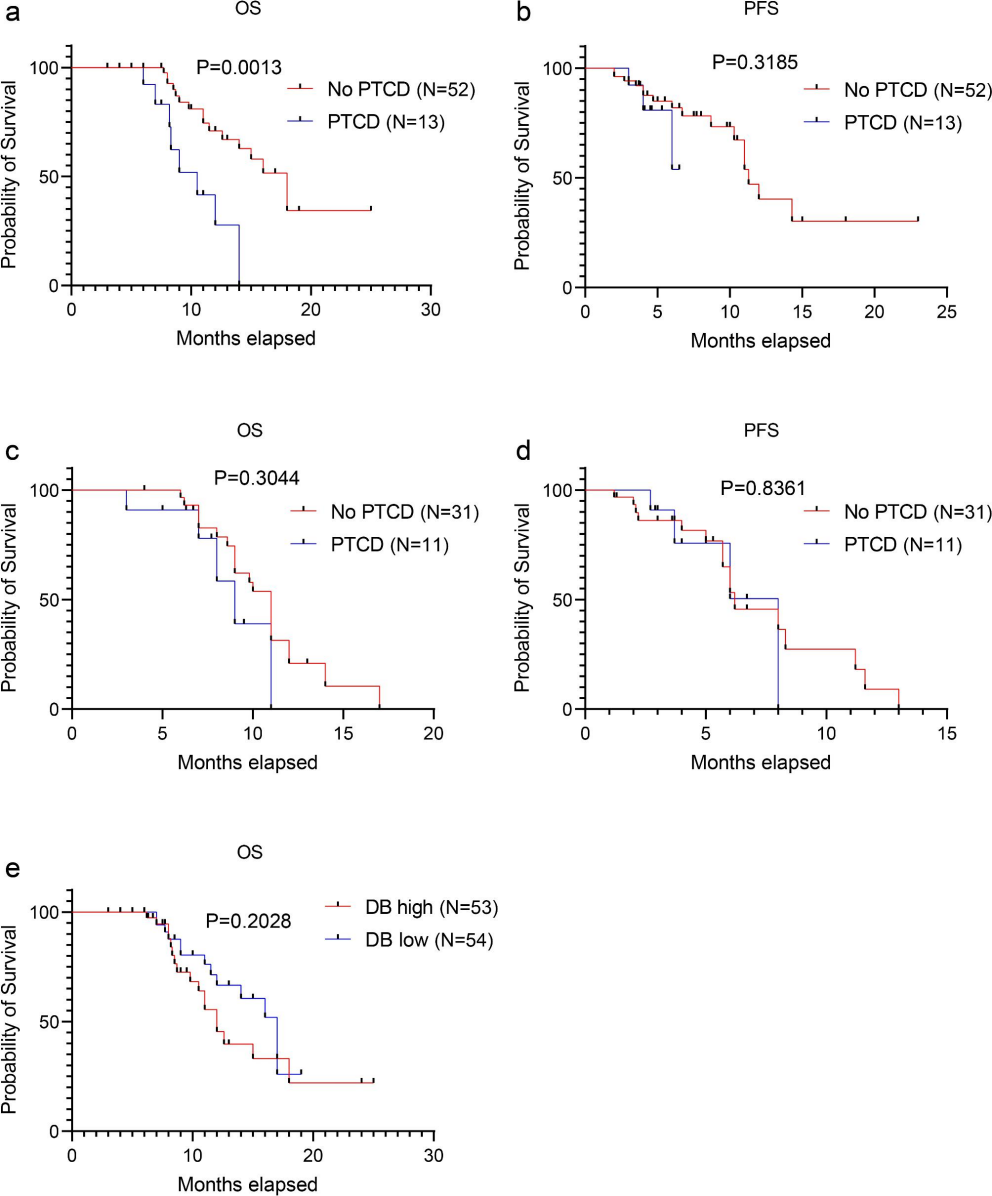
Kaplan–Meier curves of OS and PFS in patients with cholangiocarcinoma who did or not undergo percutaneous transhepatic cholangial drainage or not. immune checkpoint blockade plus chemotherapy (**a**, **b**); chemotherapy alone (**c**, **d**). The association between bilirubin concentration and OS (**e**). PTCD refers to percutaneous transhepatic cholangial drainage. DB refers to direct bilirubin

Among patients who received ICB plus chemotherapy, the median OS time for patients who underwent PTCD was 10.5 months (95% CI 7.2–13.8, *p* = 0.0013, Fig. 2a). The median PFS time for patients who did not undergo PTCD was 11.3 months (95% CI 9.8–12.8), and the median PFS time for patients who underwent PTCD was not reached (*p* = 0.243, Fig. 2b). The median OS time for patients who did not undergo PTCD was 18.0 months (95% CI 14.1–21.9), and OS time in patients who received ICB plus chemotherapy and did not undergo PTCD was significantly longer than in patients who did undergo PTCD. In the chemotherapy group, no significant difference in OS or PFS was found between patients who did or did not undergo PTCD (Fig. 2c-d). We also evaluated the association between bilirubin levels and OS and confirmed that bilirubin was not associated with worse OS in patients with CCA (*p* = 0.2028, Fig. 2e).

### Effect of ATB use on antitumor therapy

**Fig. 3:**
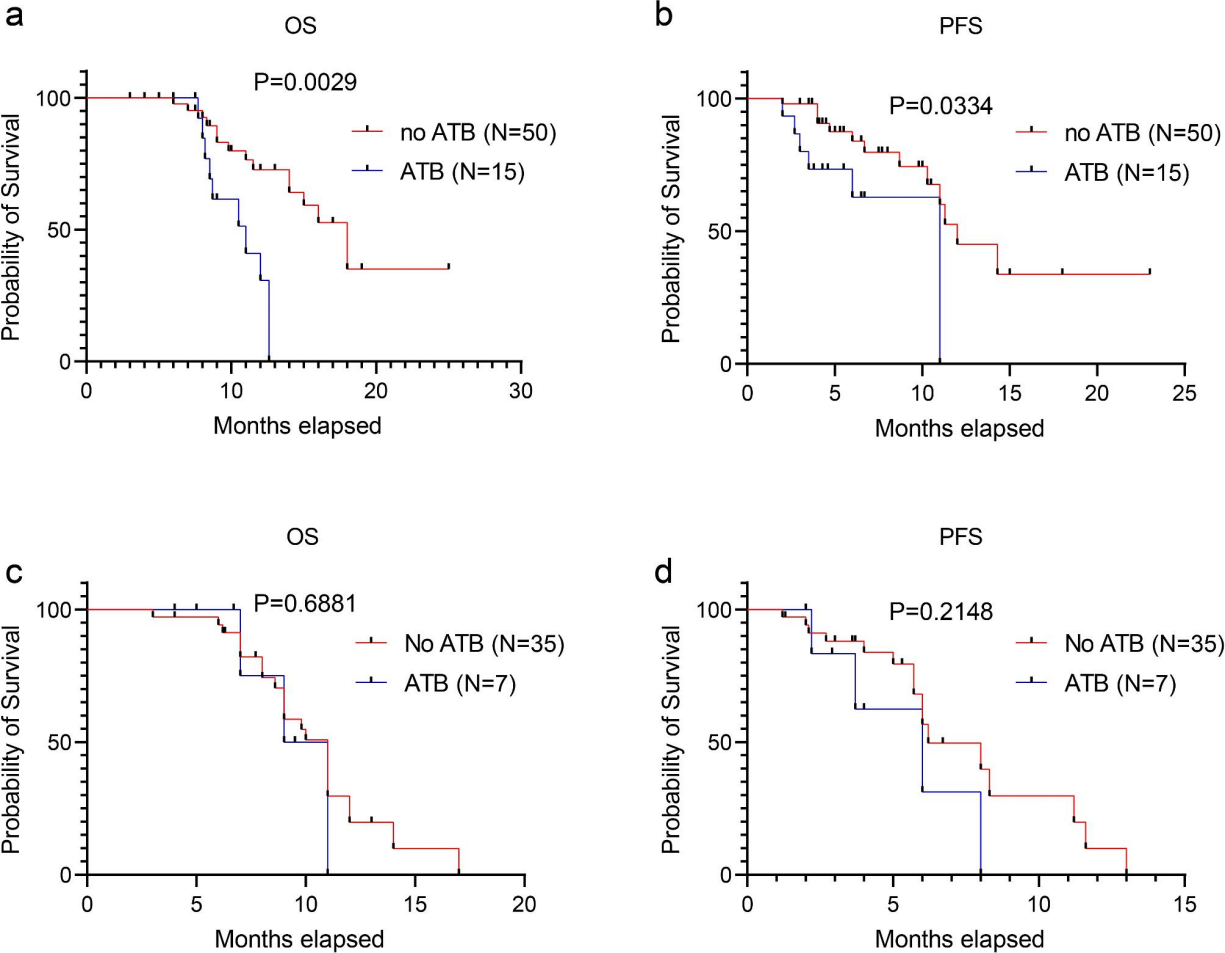
Kaplan–Meier curves of OS and PFS in patients with cholangiocarcinoma who received antibiotics. immune checkpoint blockade plus chemotherapy (**a**, **b**); chemotherapy alone (**c**, **d**). ATB refers to antibiotics

Among the ICB-plus-chemotherapy group, the median OS time and median PFS time for ATB-free patients was 18 months (95% CI 14.1–21.9) and 12 months (95% CI 8.9–15.1), respectively. The median OS time and median PFS time for patients who received ATB was 11 months (95% CI 7.7–14.3) and 11 months (95% CI, not reached–not reached), respectively (all *p* < 0.05, Fig. 3a-b). In the chemotherapy-only group, no significant differences were found in OS and PFS between patients who received ATB and those who did not (Fig. 3c-d).

### Microbiota changes in feces and bile after PTCD

**Fig. 4:**
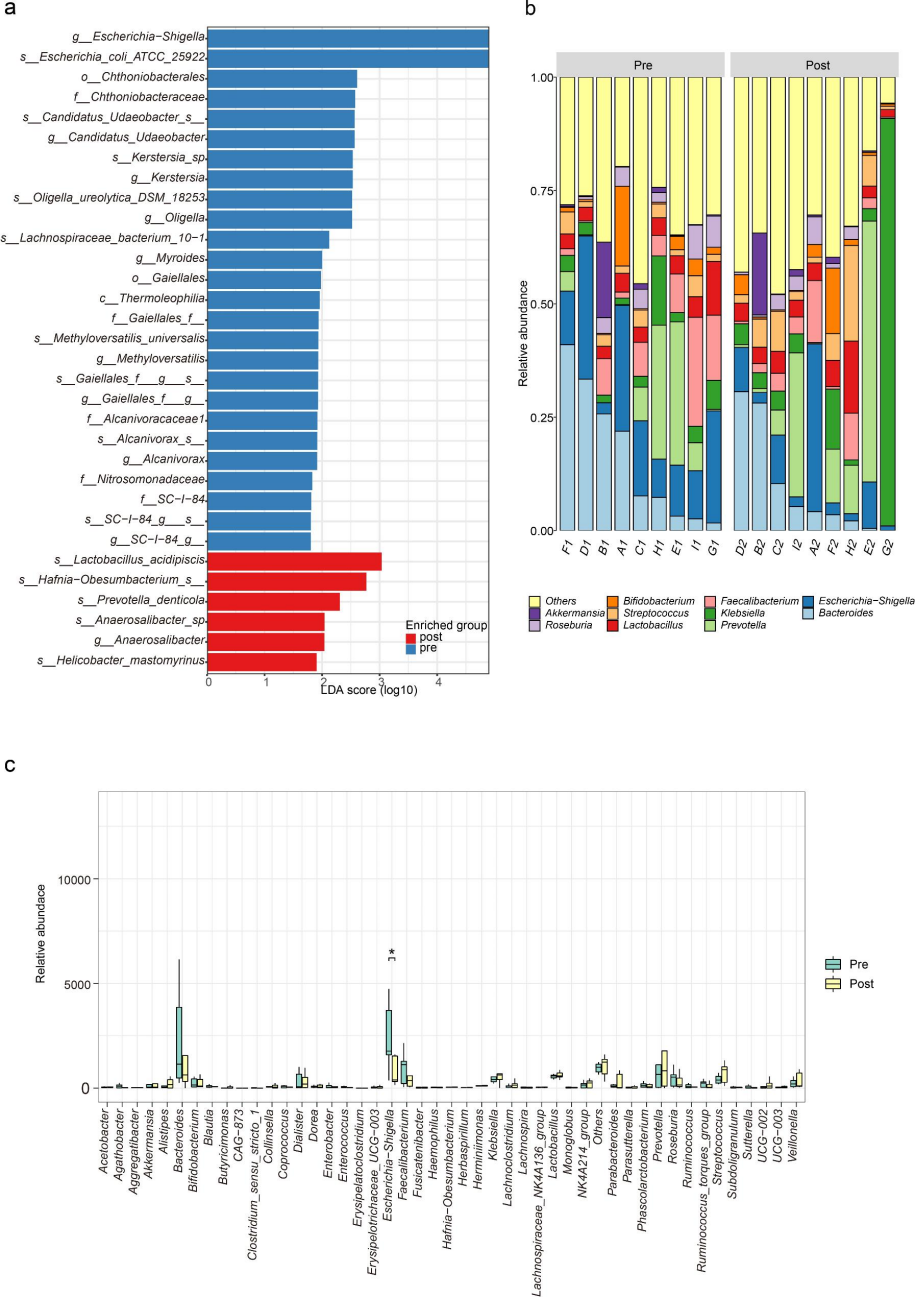
(**a**) Differential abundance analysis abundance analysis with LefSe. (**b**) Bar graph showing proportion of top10 microbes in feces pre- and post- receiving percutaneous transhepatic cholangial drainage (PTCD). (**c**) Box plots showing changes in fecal microbial abundance pre- and post- receiving PTCD. The *p*-values ​​correspond to paired Wilcoxon tests

We then sought to determine whether the microbiota composition of patients changed after receiving PTCD. LEfSe analysis determined that bacterial signatures had different compositions in fecal before and after undergoing PTCD. Before PTCD, *Escherichia − Shigella* and *Escherichia coli* predominated (Fig. 4a). In contrast, *Lactobacillus acidipiscis* and *Hafnia Obesumbacterium* predominated in patients who received PTCD 1 week later (Fig. 4a). We then analyzed the ratio of the Top10 flora in 9 patients before and after PTCD. The overall composition of the fecal microbiota was also altered after PTCD (Fig. 4b). To further identify differences in flora before and after PTCD, we subjected the Top50 flora to a Wilcoxon test and found statistically significant changes in *Escherichia-Shigella (p = 0.038)* (Fig. 4c). Collectively, these results suggest that patients had differences in gut microbiota composition before and after PTCD.

### Identification of specific genera related to changes in gut microbiota after PTCD

**Fig. 5:**
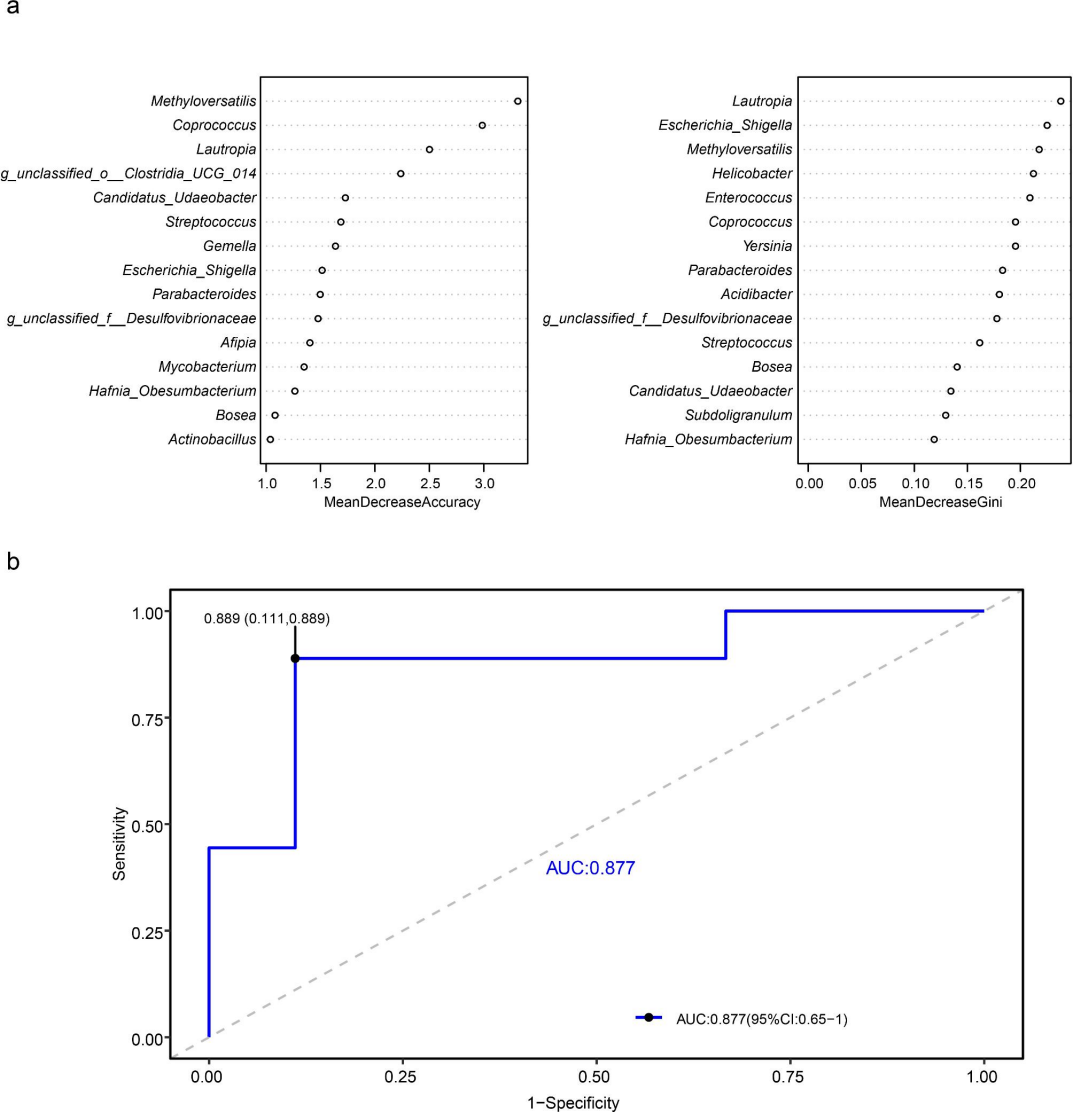
(**a**). The importance of the biomarkers was ranked according to their contribution to the Random Forest model (**b**). Receiver operating characteristic (ROC) curves and area under curve (AUC) of a random forest model based on fecal microbial

We constructed a random forest classifier model to identify those bacterial populations with the most important contributions to the differences in bacterial populations before and after PTCD, and identified *Methyloversatilis, Lautropia, Escherichia_Shigella*, and others (Fig. 5a). The area under the ROC curve for the random forest classification model was 0.877 (95% CI 0.65–1, Fig. 5b).

### Microbiota changes in bile after PTCD

**Fig. 6:**
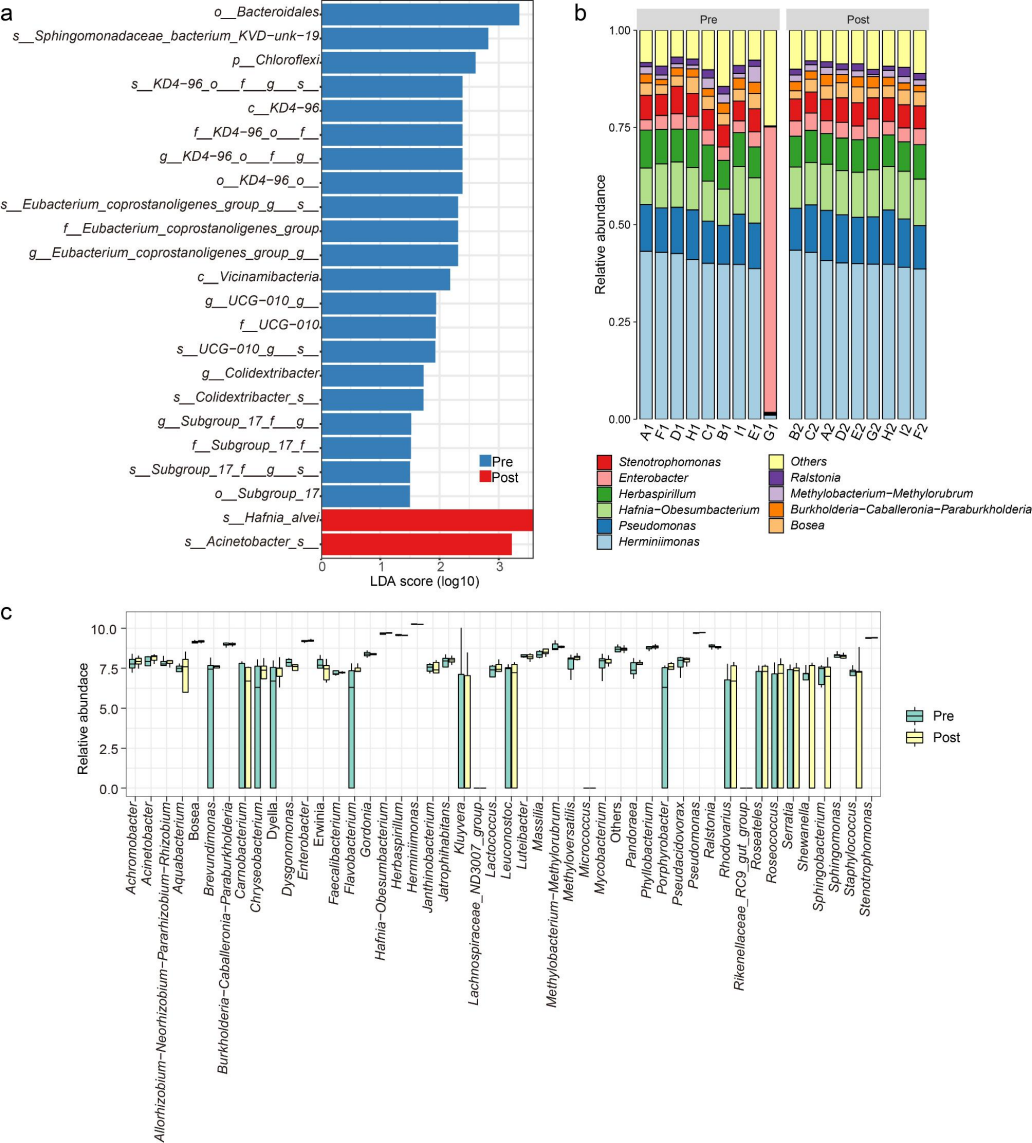
(**a**) Differential abundance analysis abundance analysis with LefSe. (**b**) Bar graph showing proportion of top10 microbes in bile pre- and post- receiving percutaneous transhepatic cholangial drainage (PTCD). (**c**) Box plots showing changes in bile microbial abundance pre- and post- receiving PTCD

LEfSe analysis of the flora enriched in bile before and after receiving PTCD revealed that *o__Bacteroidales* and *s_Sphingomonadaceae_bacterium_KVD-unk-19* were predominant in bile before PTCD, and *s_Hafnia_alvei* and *s_ Acinetobacter_s_* were predominant in bile after PTCD (Fig. 6a). We then analyzed the ratio of Top10 flora in 9 patients before and after PTCD. However, the overall composition of the bile microbiota did not change significantly after PTCD (Fig. 6b). No statistical difference was found in the Top50 flora in the bile before and after PTCD (Fig. 6c).

## Discussion

We assessed factors that might affect the effectiveness of immunotherapy, and found that CCA patients who received PTCD or ATB had a poor response to ICB plus chemotherapy and short survival time. As was true of previously published studies of several types of cancer [20–23], our study showed an association between antibiotic use and poor PFS and OS in patients with CCA. Our study was the first to evaluate the correlation between PTCD use and PFS and OS in response to immunotherapy in patients with CCA. We found that PTCD was significantly associated with worse OS, but not PFS. Although high levels of bilirubin have been linked with poor OS, we found no correlation between bilirubin and OS in our study.

The gut microbiota has been recognized as an important regulator of immunotherapy with profound effects on systemic immune responses, and dysbiosis of the gut microbiota may influence the development of cholangiocarcinoma [24–27]. Our study found that the overall composition of fecal flora was significantly changed after PTCD, specifically a decrease in *Escherichia − Shigella*, but the flora in bile did not change significantly, perhaps because of the inability of bile to enter the gut. Furthermore, the random forest classifier model built using fecal bacteria exhibited an AUC value of 0.877, indicating its accurate classification capability for bacteria before and after PTCD. This suggests significant bacterial changes associated with PTCD. Previous studies have showed that *Escherichia − Shigella* is closely related to the occurrence and development of colorectal cancer [28, 29]. Furthermore, a decreased abundance of *Shigella* in the tumor microbiota of patients with head and neck squamous cell carcinoma was found to be significantly associated with poor prognosis [30]. However, its relationship with the efficacy of immunotherapy is still unclear. Dysbiosis of the gut microbiota is common in patients with colitis [31], and clinical data show that colitis is associated with the occurrence of cholangiocarcinoma [32–34]. Zhang et al. previously reported preclinical data showing that the gut microbiota promotes the progression of CCA by increasing polymorphonuclear myeloid-derived suppressor cells to form an immunosuppressive environment [27]. This may be a mechanism by which gut microbiota affect the efficacy of immunotherapy in patients with CCA. A single-center retrospective study showed that undergoing preoperative bile drainage among patients with periampullary cancer shifted the bile microflora to a more aggressive and drug-resistant spectrum, e.g., *Enterobacter cloacae* [35]. Interestingly, some studies have linked *Enterobacterales* and *Enterobacter* with poor response to ICB across several types of cancer [36]. Another important finding of our study is that ICB plus chemotherapy had significant survival benefits in patients with CCA. As shown in the TOPAZ-1 trial, durvalumab + GemCis significantly improved OS and PFS vs. placebo + GemCis in patients with advanced CCA [37]. Unlike the TOPAZ-1 trial, most of the ICBs used in the ICB-plus-chemotherapy group in our study were PD1 blockade.

In the current study, we aimed to investigate the effects of PTCD on patients with advanced CCA whose clinical condition may have been too poor to receive the procedure. To ensure the homogeneity of our patient cohort, we used strict inclusion criteria, the most important of which was clinical status: as a result, 103 patients (96.3%) in our analysis had an ECOG score of ≤ 1 (Table 1). Cox analysis indicated that ECOG score, age, sex, and direct bilirubin were not significant risk factors for prognosis (Fig. S1). The dose, type, and timing of antibiotic use may have confounded the results. However, we did not detect any significant differences in the use of antibiotics, including their type, dose, or duration, between the chemotherapy-only versus the ICB + chemotherapy groups (Supplementary Table 3). Moreover, our Cox analysis also showed that these factors were not associated with prognostic risk factors (Fig. S2). Reasoning that ICB type may have been be a confounding factor, we also compared OS among patients using four different ICBs and found no significant differences by ICB type (Fig. S3a). Again, Cox analysis indicated that ICB type was not a prognostic risk factor (Fig. S3b).

Our study had some limitations, primarily its retrospective nature. The number of patients who underwent PTCD was small (only 24 patients), and the number of samples available for 16s rRNA sequencing (n = 9) was quite small. No further experiments were done to verify the differences in bacteria, but we are planning to address this in the future. Our results indicate that immunotherapy may benefit patients with advanced CCA. Notably, PTCD was found to attenuate the effect of immunotherapy on bile duct cancer. We plan to validate our findings with a larger sample in a multicenter clinical cohort.

## Conclusions

The combination of ICB and chemotherapy improved survival in patients with CCA, particularly in those who do not undergo PTCD. Moreover, PTCD seems to alter the composition of gut microbiota, but not bile microbiota.

## Electronic supplementary material

Below is the link to the electronic supplementary material.

Supplementary Material 1

## Data Availability

The data that support the findings of this study are openly available in National Center for Biotechnology Information at https://www.ncbi.nlm.nih.gov/, with the reference number as PRJNA972988 and PRJNA973004.

## Abbreviations

PTCD: Percutaneous transhepatic cholangial drainage
ATB: Antibiotic
OS: Overall survival
PFS: Progression-free survival
CCA: Cholangiocarcinoma
ICB: Immune checkpoint blockade
