# Supplementary material for: Percutaneous transhepatic cholangial drainage or antibiotic therapy worsens response to immunotherapy in advanced cholangiocarcinoma
Source: BMC Cancer. 2023 Jul 13;23:657. doi: 10.1186/s12885-023-11128-2 (PMC10347737; doi:10.1186/s12885-023-11128-2)
Supplement: Supplementary file 1 — Supplementary Material 1 [file 12885_2023_11128_MOESM1_ESM.docx]

**Supplementary Table 1** Multivariate survival analysis for all patients

| Variables | Overall survival | | Progression-free survival | |
| --- | --- | --- | --- | --- |
|  | P value | HR (95% CI) | P value | HR (95% CI) |
| Age (years) |  |  |  |  |
| ≥60 vs. <60 | 0.127 | 1.022 (0.994-1.050) | 0.924 | 1.001 (0.969-1.035) |
| Sex |  |  |  |  |
| Male vs. Female | 0.823 | 1.071 (0.588-1.951) | 0.952 | 1.080 (0.517-2.015) |
| ECOG score |  |  |  |  |
| 0-1 vs. 2 | 0.981 | 46089.917 | 0.290 | 0.326 (0.041-2.597) |
| Treatment |  |  |  |  |
| ICB+ Chemo vs. Chemo | 0.000 | 0.340 (0.187-0.619) | 0.381 | 0.354 (0.198-0.735) |
| PTCD |  |  |  |  |
| Yes vs. No | 0.003 | 2.840 (1.443-5.592) | 0.004 | 1.238 (0.612-3.617) |

ECOG, Eastern Cooperative Oncology Group; ICB, immune checkpoint blockade; PTCD, percutaneous transhepatic cholangial drainage; Chemo, chemotherapy

**Supplementary Table 2** Multivariate survival analysis for patients receiving ICB+Chemo

| Variables | Overall survival | | Progression-free survival | | |
| --- | --- | --- | --- | --- | --- |
|  | P value | HR (95% CI) | P value | HR (95% CI) |  |
| Age (years) |  |  |  |  |  |
| ≥60 vs. <60 | 0.089 | 1.036 (0.995-1.079) | 1.001 | 1.001 (0.958-1.046) |  |
| Sex |  |  |  |  |  |
| Male vs. Female | 0.780 | 0.879 (0.356-2.172) | 0.798 | 0.798 (0.296-2.429) |  |
| ECOG score |  |  |  |  |  |
| 0-1 vs. 2 | 0.986 | 19883.768 | 0.233 | 0.251 (0.026-2.429) |  |
| PTCD |  |  |  |  |  |
| Yes vs. No | 0.002 | 4.321 (1.702-10.969) | 0.259 | 2.208 (0.558-8.740) |  |

ECOG, Eastern Cooperative Oncology Group; PTCD, percutaneous transhepatic cholangial drainage

**Supplementary Table 3** Use of antibiotics

|  |  | Chemotherapy | ICB+chemotherapy | *P* value |
| --- | --- | --- | --- | --- |
| TYPE |  |  |  |  |
| Quinolones |  | 1 | 3 | 0.746 |
| β-lactams |  | 6 | 12 |  |
| Dose |  |  |  |  |
| ≥40.5g |  | 2 | 8 | 0.277 |
| ＜40.5g |  | 5 | 7 |  |
| Duration |  |  |  |  |
| ≥7 Days |  | 4 | 6 | 0.452 |
| ＜7 Days |  | 3 | 9 |  |

Statistically Significant for Fisher’s exact test; ICB, immune checkpoint blockade

**Fig. S1
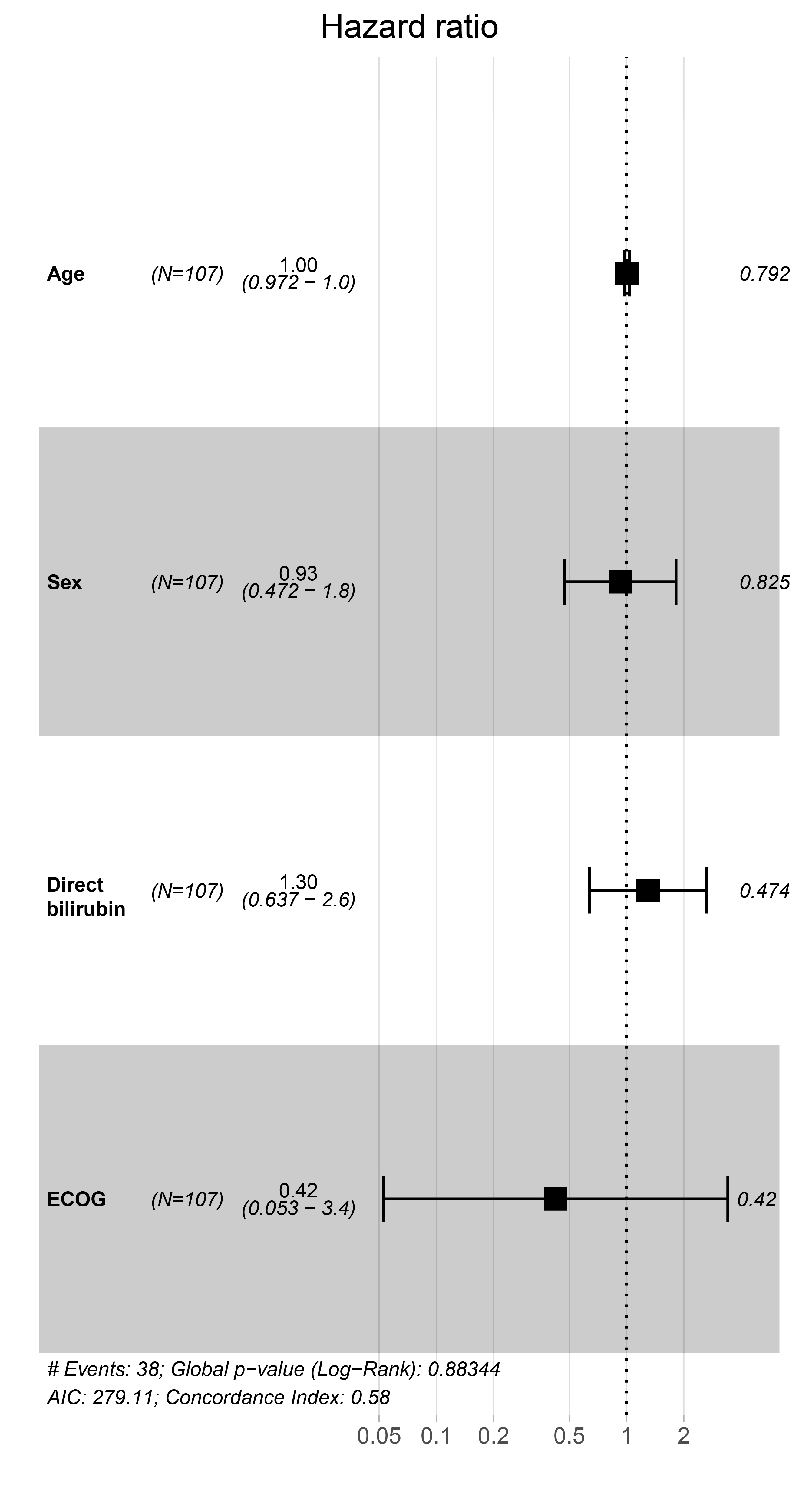
**


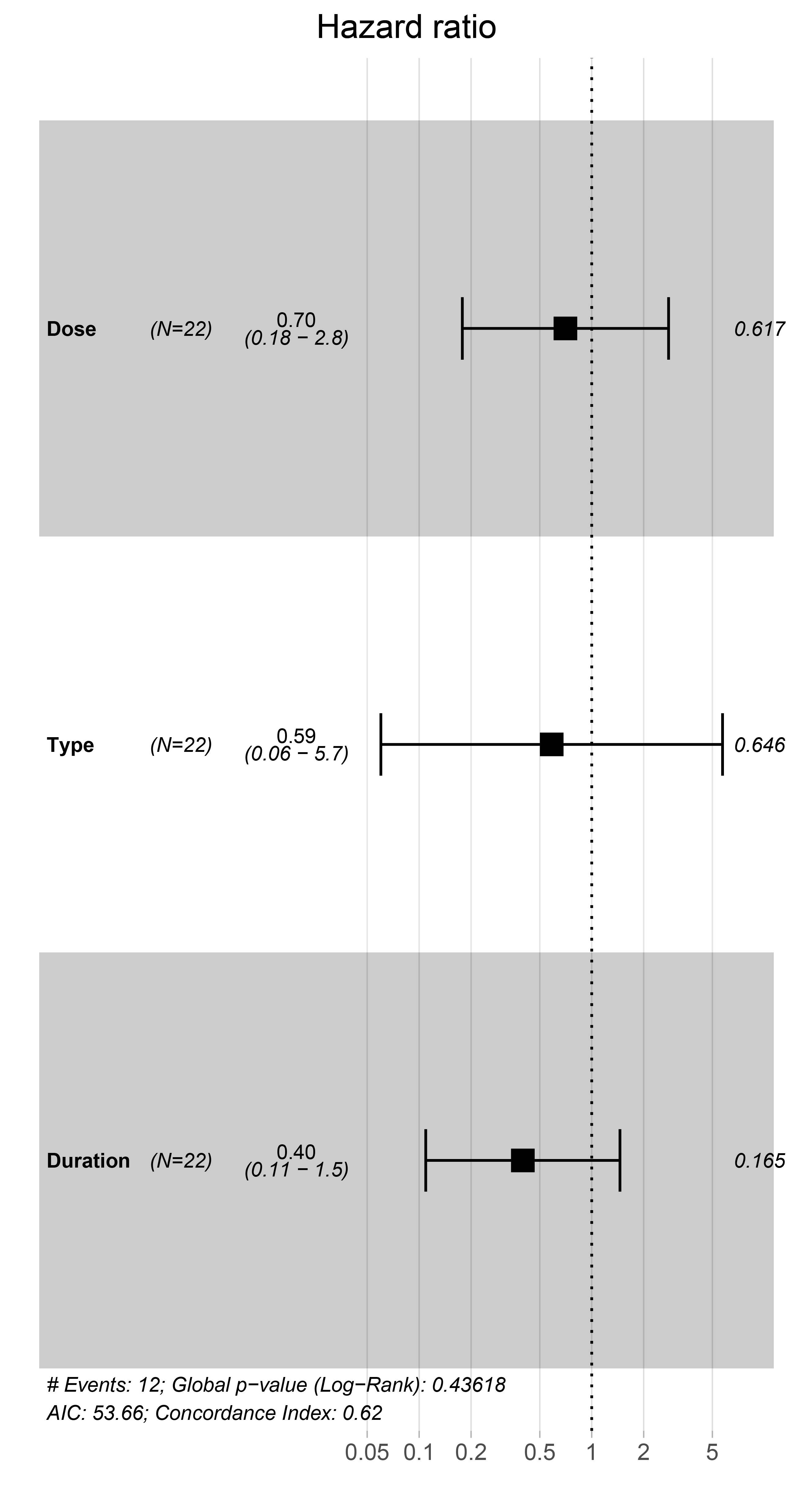
**Fig. S2**

**
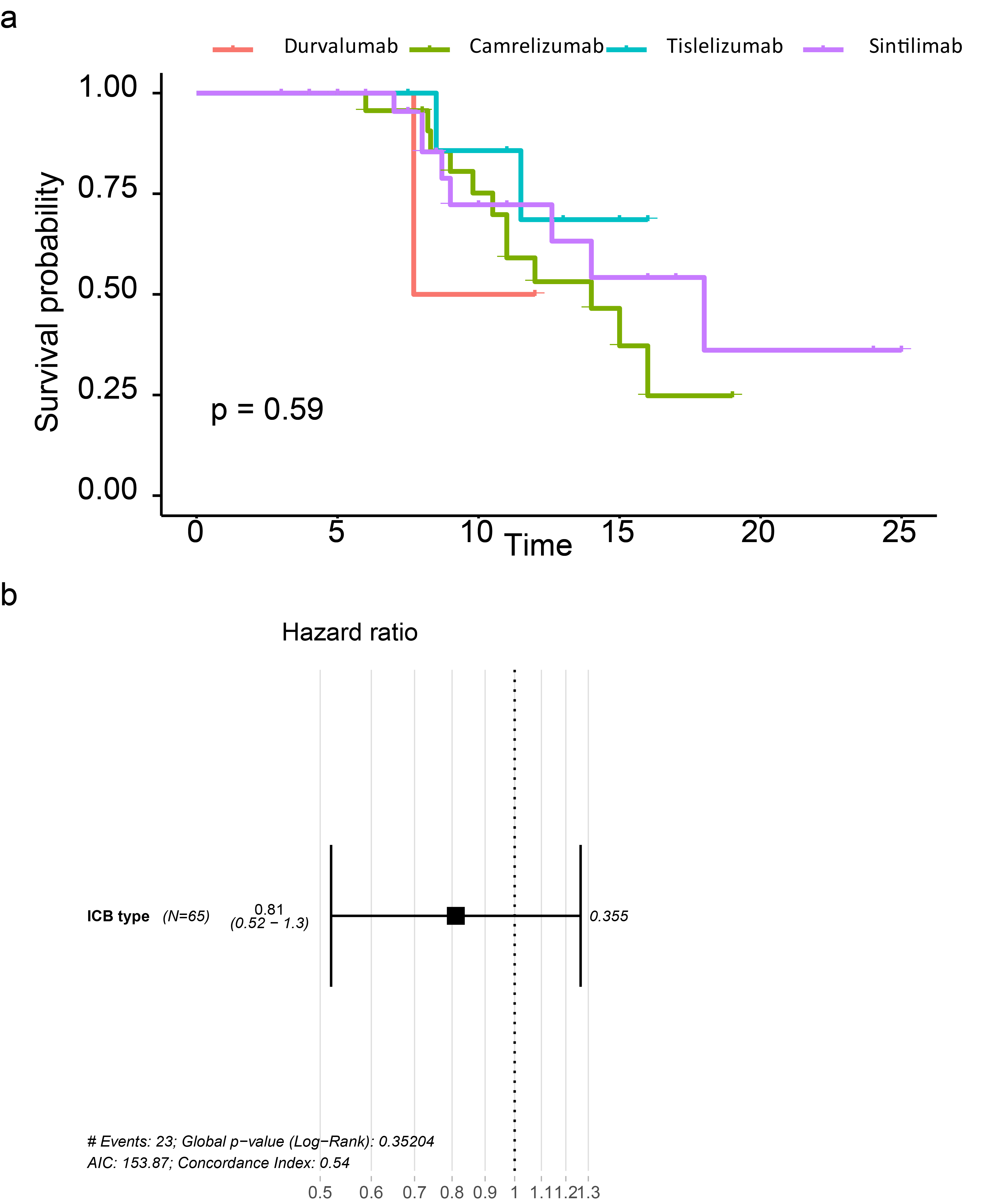
Fig. S3**

**Figure captions**

**Fig. S1** Cox analysis of OS for all patients.

**Fig. S2** Cox analysis of OS in patients receiving antibiotic therapy

**Fig. S3** (a) Overall survival of four ICB, (b) Cox analysis of OS in patients receiving ICB + chemotherapy group. ICB, immunotherapy checkpoint blockade
